# An energy-aware protocol in wireless sensor networks using the scattered search algorithm and fuzzy logic

**DOI:** 10.1371/journal.pone.0297728

**Published:** 2024-11-04

**Authors:** Shayesteh Tabatabaei

**Affiliations:** Department of Computer Engineering, University of Saravan, Saravan, Iran; The Islamia University of Bahawalpur Pakistan, PAKISTAN

## Abstract

Given the resource limitations of wireless sensor networks (WSNs), energy conservation is of utmost importance. Moreover, minimizing data collection delays is crucial to maintaining data freshness. Additionally, it is desirable to increase the number of collected data samples to enhance accuracy and robustness in data collection. For this purpose, this research article proposes a clustering-based routing protocol aimed at maximizing the delivery of data samples while minimizing energy consumption and data collection delays. The protocol employs a scattered search algorithm and fuzzy logic to cluster the sensor nodes. By considering the distance to the sink and the remaining energy level of the battery, the network is dynamically divided into clusters using a lightweight clustering approach. To evaluate the effectiveness of the proposed method, simulations were conducted in OPNET using the AFSRP protocol. The results demonstrate superior performance of the proposed method in terms of end-to-end delay by 13.44%, media access delay by 75.2%, throughput rate by 20.55%, energy consumption by 13.52%, signal-to-noise ratio by 43.40% and delivery rate of successfully sending data to the sink is 0.21% higher than the well-known AFSRP method.

## 1. Introduction

A WSN is a network comprising sensor nodes that collaborate to accomplish a common task. Each sensor node comprises various models, including a processor model, a sensor model, and a communication model [1]. The sensor model is responsible for measuring parameters such as pressure, temperature, motion, and so on. The measured values are then transmitted to a central point known as the sink. WSNs find applications in diverse fields, including healthcare monitoring, gaming, and entertainment, as well as environmental and military domains. Despite their versatility, sensor nodes face multiple constraints, such as unreliable communication links and limited battery energy. Moreover, in certain applications, such as military environments, sensor nodes may remain inaccessible for extended periods, making battery replacement highly challenging, if not impossible. Consequently, it is imperative to design an energy-efficient communication protocol for WSNs. Based on the network architecture, WSNs can be categorized into distributed (flat) networks and hierarchical networks. In a flat network, homogeneous sensor nodes with similar responsibilities are employed. A self-organizing network is formed by randomly deployed nodes, all executing the same protocol, program, and code. In a flat topology network, it is common for nodes to avoid establishing direct communication with the sink to prevent premature depletion of energy in nodes located farther away from the sink, which can result in a decrease in the overall network size. To address this, multi-hop communications are utilized to reduce energy consumption for long-distance transmissions at remote nodes. However, the implementation of multi-hop communications introduces various challenges that significantly impact the network’s design and performance.

One significant challenge arises from the nodes located around the sink. These nodes experience faster energy depletion compared to other nodes since they not only transmit their own data but also relay network traffic. Consequently, the failure of these nodes can lead to the isolation of the sink from the rest of the network. This issue is commonly referred to as the hot spot problem or hotspot. The energy consumption in flat topologies is unevenly distributed, resulting in smaller network sizes in direct communication scenarios and the detachment of network nodes from the sink in multi-hop scenarios. As a result, the flat architecture is more suitable for small-scale networks.

Consequently, MAC protocols designed for flat-topology WSNs are not efficient in terms of energy consumption. To address these limitations, researchers have proposed clustering algorithms as a solution for flat-topology networks. It has been widely suggested that a suitable clustering approach can effectively reduce energy consumption in WSNs by minimizing energy consumption in data transmission and the number of transmitted packets [2]. To tackle energy management in WSNs, a range of protocols and communication methods have been proposed. These solutions take into account various clustering techniques [3] as their foundational concepts. The network coverage area is partitioned into smaller regions known as clusters. Within each cluster, a specific node is designated as the Cluster Head (CH), assuming the responsibility of managing both intra-cluster and inter-cluster communications. Non-CH nodes within a cluster collect and transmit the gathered information to their respective CHs. Once the CH receives data from cluster members, it proceeds to forward the aggregated results either to the next CH using multi-hop routing or directly to the sink [4].

The utilization of clustering is a beneficial and efficient approach to decreasing energy consumption and extending the lifespan of WSNs [4]. This research article introduces a novel approach utilizing a scatter search algorithm and fuzzy logic for effective clustering and CH selection in sensor networks. The scatter search algorithm is an optimization algorithm used to solve complex optimization problems. This algorithm operates based on group search, simultaneously exploring multiple search points in the search space. The main advantage of the scatter search algorithm lies in its ability to find optimal solutions in the search space. It can perform multiple optimizations and approach both optimal and near-optimal solutions. On the other hand, fuzzy logic is a mathematical method that aids in dealing with uncertainty and ambiguity in scientific and engineering achievements. fuzzy logic uses fuzzy sets and fuzzy members for more accurate and stable modeling and description of classifiers. This method is typically employed in situations where precise and definite criteria for decision-making are lacking. The motivation behind the proposed method is to combine the scatter search algorithm with fuzzy logic. By integrating the scatter search algorithm with fuzzy logic, a robust and efficient approach for solving complex optimization problems such as clustering can be achieved. The scatter search algorithm can be considered a tool for searching in a search space. By utilizing a combination of multiple independent and scattered solutions, the scatter search algorithm reduces the likelihood of getting stuck in local minima. Fuzzy logic can be employed to provide a more accurate description and logical modeling of the fitness function of the sensor nodes used. In essence, fuzzy logic can handle uncertainty and ambiguity in the selection of suitable nodes. Therefore, the combination of scatter search with fuzzy logic enables the optimization algorithm to improve its performance and achieve better and more acceptable solutions for selecting appropriate CHs. The primary objective of this method is to minimize energy consumption and enhance the lifespan of WSNs. In this proposed approach, the traditional single-hop communication between CHs and the sink is replaced with an optimized multi-hop communication scheme. Furthermore, the method calculates the optimal number of clusters and optimizes energy consumption by dividing the network into categories with similar sizes, resembling a distributed configuration.

This paper introduces several key contributions:

- Introducing an innovative clustering approach that combines the scatter search algorithm with fuzzy logic to determine the optimal CH.
- Formulation of a mathematical model for the fitness function based on two distinct features, battery energy and distance from the sink, utilizing fuzzy logic.
- Proposal of a fuzzy model to mitigate uncertainties associated with obtaining probability values for each node, facilitating the selection of the most suitable node as the CH.
- Formulation and implementation of an energy-centric and distance-centric clustering algorithm based on stabilized fuzzy input parameters related to energy and distance. The algorithm aims to minimize energy consumption across nodes in WSNs.
- To achieve a balance between energy consumption and data transmission delay, an adaptive multi-hop cluster-tree routing structure is employed for each cluster.
- Comparative analysis of the proposed method with the AFSRP method.
- The versatility of the approach is not confined solely to the mentioned two parameters. It can be applied to various other commonly used naive fuzzy input parameters for clustering in WSN, thereby enhancing the performance of existing models.

The subsequent sections of this paper are organized as follows: The second section provides an overview of related research on enhancing energy consumption in wireless sensor network (WSN) networks. The third section presents the scatter search algorithm and fuzzy logic utilized for WSN clustering. The fourth section presents the proposed method and simulation results conducted using the OPNET simulator. Finally, the fifth section presents the conclusion of the study.

## 2. Related works

Numerous investigations in the domain of WSNs have illustrated that employing a clustering protocol is a beneficial strategy for achieving load balancing and enhancing network longevity. However, devising an optimal clustering technique for WSNs poses a complex computational challenge, known as an NP-hard problem, which can be effectively tackled through the utilization of soft computing techniques. As a result, significant research efforts have been dedicated to exploring this area. In a recent study [5], a novel approach called FFA-RF was proposed for clustering in WSNs. The FFA-RF protocol combines the Firefly Algorithm (FA) and Fuzzy-Firefly Algorithm (FFA) with the Random Forest (RF) technique. The protocol consists of two main stages: offline configuration and online routing. During the offline stage, an optimized fuzzy inference system (FIS) based on FFA is developed. This system is applied to various network topologies, generating a comprehensive dataset. The dataset is then utilized to train and test an RF model.

In the online stage, the trained RF model serves as an online clustering algorithm. It estimates fuzzy priority coefficients for nodes, such as determining continuation elimination or direction selection for CHs, in new network instances. The FFA-RF model is subsequently tested on real network samples. Simulation results demonstrate the superior performance of the FFA-RF protocol compared to crisp heuristic, fuzzy heuristic, metaheuristic, and combined fuzzy-metaheuristic protocols in terms of network lifetime.

The Firefly Algorithm (FFA) operates as a metaheuristic algorithm, utilizing the stochastic movements of fireflies to explore the solution space. However, this approach may result in drawbacks, such as being trapped in local optima. Additionally, the Random Forest (RF) is a supervised learning algorithm, necessitating labeled data for classifier training. This reliance on labeled data may constrain the method’s applicability within WSNs, but the proposed method in this paper uses the scatter search algorithm as an unsupervised learning algorithm. to find optimal solutions in the search space. It can reduce the likelihood of getting stuck in local minima.

A recent study [6] introduced a novel optimized CH selection method based on the genetic algorithm (NCOGA) and a novel clustering technique for WSNs based on the genetic algorithm. NCOGA incorporates adaptive crossover and tournament selection techniques to enhance the network’s longevity. The key innovation of this method lies in the integration of multiple parameters for selecting CHs in a heterogeneous WSN. In NCOGA, the fitness parameter is computed by combining various factors, including residual energy, initial energy, distance to the sink, number of node neighbors, load balancing factor, and Communicating Mode Decider (CMD). The load balancing and CMD parameters serve three objectives: identifying the optimal candidate node as the CH, determining the communication mode (single-hop or multi-hop) of the CH, and preventing hotspots within the network. An important characteristic of the proposed method is that it exclusively considers nodes with energy levels exceeding the threshold energy. This feature enables NCOGA to converge towards the optimal solution more efficiently. Through simulations, the results demonstrate that the proposed protocol outperforms advanced optimization algorithms based on the genetic algorithm in terms of stability period, residual energy, network lifetime, and operational power.

The proposed method in [6] utilizes six distinct functions based on six criteria to measure fitness, resulting in a high computational load. In contrast, the proposed method in this paper employs only two criteria for the performance measurement function, aiming to streamline computational complexity.

In a recent study [7], an energy-aware clustering method called Quantum Tunicate Swarm Algorithm Based Energy Aware Clustering (QTSA-EAC) was introduced for WSNs. The primary goal of this method is to efficiently manage energy distribution among the network nodes, thereby maximizing the WSN’s lifetime.

QTSA-EAC is derived from the integration of quantum computing principles with the traditional Tunicate Swarm Algorithm (TSA). By incorporating multiple parameters, the proposed method aims to select CHs in a manner that promotes energy efficiency and extends the WSN’s lifespan. Performance evaluations of QTSA-EAC demonstrate its effectiveness in improving network lifetime, reducing energy consumption, and achieving lower latency compared to alternative approaches.

The algorithm uses quantum bits to represent the solutions and applies quantum operators to update them. This may introduce complexity and computational overhead to the algorithm, especially for large-scale WSNs. Additionally, the fitness function is defined as a weighted sum of four criteria: residual energy, distance to the base station, distance to the CH, and cluster size. The paper lacks a clear justification or analysis regarding the determination of these weights and their impact on the algorithm’s performance. The approach described in this paper incorporates two criteria, namely the distance to the sink and the remaining energy level of the battery, within the fitness function. Additionally, it integrates fuzzy logic to address uncertainties and ambiguities in the selection of appropriate nodes. As a result, it exhibits lower complexity and computational overhead compared to the method proposed in reference [7].

In cluster-based sensor networks, data transmission from a CH to the sink node can be accomplished through other CHs using a multi-hop approach. Consequently, two crucial issues, namely CH selection and optimal multi-hop routing, must be addressed. To address these challenges, a novel method based on optimal CH selection and optimal multi-hop routing is proposed in [8] to enhance the network’s longevity. The proposed scheme utilizes a genetic algorithm at two levels. At the first level, the genetic algorithm selects the CHs, while at the second level, it determines the optimal multi-hop routing among them. Simulation results of the proposed method demonstrate a significant enhancement in the network’s lifetime.

The paper [8] does not provide a clear definition or explanation of the network model, the energy consumption model, or the objective function that is used in the optimization problem. This makes it difficult to understand the assumptions and constraints of the proposed approach and to compare it with other existing methods. Also, it uses a 2-level genetic algorithm, which involves multiple iterations of selection, crossover, and mutation operations. This may require a lot of computation and communication resources, which may not be feasible or efficient for resource-constrained sensor nodes, but the proposed method in this paper provides a clear definition of the objective function, network model, and the number of iterations.

A novel routing approach is introduced in [9] to tackle the challenges associated with clustering in WSNs, including fault tolerance, load balancing, and the attainment of optimal local solutions. This technique encompasses two stages: optimal CH selection and data transfer. The optimal CH selection stage employs the Moth Levy-adopted Artificial Electric Field Algorithm (ML-AEFA). Several criteria are considered in the selection process, including energy levels, node degree, sensor node distance, distance between CHs and the base station (BS), as well as node death time. The data transfer stage relies on customized gray-wolf optimization (CGWO). This stage facilitates efficient data transmission within the network. Through simulations, it is demonstrated that the proposed method significantly enhances the network lifetime. Specifically, for a hundred sensor nodes, the network lifetime increases by approximately 35.77%, 35.77%, 35.04%, 34.43%, and 33.08% compared to GWO, MSA, AEFA, BOA, and + ACO, respectively.

The paper [9] uses two complex optimization algorithms, which involve multiple iterations of random search, levy flight, electric field calculation, and grey wolf update. This may require a lot of computation and communication resources, which may not be feasible or efficient for resource-constrained sensor nodes.

Also, the Gray Wolf Optimization algorithm may experience low genetic diversity and get trapped in local optima during the early stages of execution. Additionally, the electric field calculation algorithm involves mathematical complexities arising from integrations, coordinate transformations, or solving differential equations. However, the suggested approach in this paper avoids falling into local optima due to the use of the scatter search algorithm and exhibits lower mathematical computational complexity compared to this method.

A novel energy-aware clustering technique for data transmission in WSNs is introduced in [10]. The proposed method builds upon the LEACH protocol and integrates the Harmony Search Algorithm (HSA) with Competitive Swarm Optimization (CSO) for CH selection. The CHs are selected based on their energy levels, employing a global search approach that exhibits a fast convergence rate. The proposed method demonstrates high efficiency in the search process, capitalizing on the strong performance of the HSA search and the dynamic capabilities of CSO. As a result, it enhances the lifetime of sensor nodes. In terms of throughput rate and remaining energy, the combined HSA-CSO algorithm outperforms existing methods, delivering superior performance.

In [10], the Harmony Search algorithm is heavily dependent on numerous parameters that significantly impact its performance. These parameters include the harmony memory size, pitch adjustment rate, and pitch changing rate. Optimal selection of these parameters is a challenge. Additionally, the Harmony Search algorithm relies solely on harmony memory to generate new solutions and lacks random jumps. Consequently, this algorithm cannot definitively guarantee finding the optimal solution. Similarly, the Competitive Swarm Optimization algorithm also cannot unequivocally guarantee discovering the optimal solution. However, the proposed method in this paper, by integrating fuzzy logic and scatter search algorithm, demonstrates the capability to find optimal solutions.

In [11], a novel approach combining Butterfly Optimization (BOA) and Ant Colony Optimization (ACO) is proposed to address energy consumption, network lifetime, and hot spot issues in wireless sensor network clustering. The method encompasses two scenarios: a hybrid butterfly and ant colony system with a static sink node (HBACS) and a hybrid butterfly and ant colony system with a mobile sink node (HBACM). BOA is leveraged to identify the optimal CH, resulting in reduced network energy consumption, alleviated CH load, and efficient exploration of solutions within the WSN. The Static Sink Node (SSN) is strategically deployed in multiple positions (center, corner, and outside) within the area of interest. Additionally, a mobility model and mobility function are employed to position the Mobile Sink Node (MSN), enabling data collection and addressing hot spot concerns. ACO facilitates energy-efficient routing, minimizing energy consumption, and extending the network’s lifetime. Simulation results demonstrate improvements in remaining energy, the number of live nodes, and operational power for the HBACS and HBACM methods compared to the CRWO, ERP, and IHSBEER algorithms.

In [13], a novel approach called Optimal Clustering in Circular Networks (OCCN) is introduced for efficient clustering in circular sensor networks. This method employs a systematic process to determine the optimal number of clusters based on the one-hop intra-cluster communication distance. OCCN divides the network range into multiple concentric rings, with each ring surrounding the sink. The distance between the boundaries of consecutive rings is set to the appropriate distance between two neighboring nodes in a multi-hop communication scenario. Each ring comprises multiple clusters based on its designated range, and the suitability of clustering within these clusters is evaluated. One notable advantage of OCCN is its scalability, as it can be implemented using multi-hop communication. By minimizing energy consumption, OCCN effectively extends the network’s lifetime.

In the paper [13], CHs are exclusively selected based on the energy level criterion, neglecting essential metrics such as the distance to the sink. Furthermore, due to the hierarchical transmission of all packets to the CHs closer to the sink, these nodes bear a higher communication load, resulting in quicker battery depletion and a hot-spot problem.

In contrast, the proposed method in this paper considers both the distance to the sink and the energy level of the battery as criteria for CH selection. Additionally, each CH, if located within the sink’s range, directly transmits its data.

In [14], a distance-based energy-efficient algorithm is proposed to enhance energy consumption in WSNs. The algorithm introduces a mechanism where the sink node establishes new neighboring nodes each time it relocates within the network. Each neighboring node calculates its distance to the sink and transmits data only if the distance falls within an acceptable range. However, if the distance exceeds a specific threshold, an auxiliary node is employed to assist with data transmission. Following data transmission, the mobile sink proceeds towards its next deployment location, sending two messages—namely, the arrival message and the next-position message—to its neighbors upon arrival. It is important to note that no data exchange occurs between location 1 and location 2 during the relocation period of the sink. Simulation results indicate that a network utilizing multiple mobile sinks outperforms a network with a single sink in terms of overall network performance. Furthermore, the deployment of three mobile sinks in the network contributes to an extended network lifetime and a substantial reduction in energy consumption.

In paper [14], if the distance exceeds a specific threshold, an auxiliary node is employed to assist with data transmission.

An auxiliary node, due to transferring the load of other nodes, bears a high energy burden, depleting its energy and disrupting the network topology. In the proposed method in this paper, CHs are selected based on both energy levels and distance, and only if a CH is not within the sink range can it utilize other CHs for data.

In [15], a novel approach for CH selection in WSNs is introduced. The proposed method involves a series of steps to effectively determine the CHs within the network. Initially, CHs are chosen randomly, and regular nodes send join messages to express their interest in becoming a member of a cluster. Additionally, the regular nodes transmit their remaining energy to the temporary CH. The temporary CH then computes the total remaining energy of the network based on the energy levels of the nodes. If the remaining network energy exceeds a certain threshold (x%), the node with the highest energy is designated as the CH. However, if the remaining network energy falls below the threshold, the node with the highest number of neighbors is selected as the new CH. The proposed method takes into consideration both energy and location criteria for CH selection, leading to an increased network lifetime.

In the paper [15], only the criterion of remaining energy is considered for cluster selection, and other metrics such as distance to the sink are not taken into account. In contrast, the proposed method in this paper considers both criteria for cluster selection.

In [16], a novel routing protocol named CBA (cluster-based client/server data aggregation) is introduced. This protocol presents a dynamic clustering mechanism that divides the network into data-centric clusters using a lightweight approach based on the Hamming distance. The CHs within each cluster establish a minimum spanning tree (MST) to serve as the network’s backbone, facilitating the transmission of aggregated results to the sink. To minimize the overhead associated with creating the tree infrastructure, a parallel collision avoidance technique is employed. Through simulations, it is demonstrated that the proposed method significantly reduces energy consumption, decreases data aggregation delay, and improves the accuracy of data sampling.

In the paper [16], only the Hamming distance criterion is considered for selecting CHs, while the proposed method also takes into account the criterion of battery level energy.3. Suggested method.

In the paper [17], a novel approach was presented to tackle the challenge of optimizing energy consumption in WSNs. They introduced an energy-aware routing protocol named AFSRP, utilizing a congestion optimization algorithm inspired by the swarm intelligence of fish. This protocol aims to enhance energy efficiency in these networks. The proposed protocol underwent simulation against the ERA protocol in the OPNET 11.5 simulator, revealing its superior performance in terms of energy consumption, end-to-end delay, access delay, throughput rate, success probability of transmission to the sink, and signal-to-noise ratio compared to the ERA protocol. However, a drawback of this approach is its optimization of fish in static environments, and its convergence speed may decrease in dynamic environments such as sensor networks. The method introduced in this paper, incorporating scatter search and fuzzy logic, is specifically tailored for dynamic environments.

In the paper [18], a clustering protocol for WSNs is presented. The protocol, known as centralized genetic-based clustering (CGC), utilizes a genetic-based strategy combined with the incorporation of the "onion" technique. The main goal of this method is to improve the effectiveness and performance of sensor networks through the use of genetic algorithms in the clustering process. By having a structure, this protocol systematically facilitates the creation of clusters within the network. Additionally, the paper introduces an onion methodology to segment the network into layers and reduce communication burdens among CH nodes. One limitation of this approach is that as problem complexity grows, scalability issues may arise with algorithms, leading to higher computational expenses for large-scale optimization challenges. In contrast, the proposed approach in this study results in reduced processing.

Paper [19] introduces a cluster-based routing method using a mobile sink in WSNs. This paper aims to enhance the network’s lifetime, energy consumption, end-to-end delay, and packet delivery ratio by considering the role of CHs in traffic transfer between clusters and controlling the movement of sinks. In the proposed method, nodes are initially clustered based on their scores, and then the network is divided into sections, each assigned a mobile sink moving along a predefined path to collect data from the CHs. One drawback of this approach is that determining the optimal path for the sink’s movement in the network may be complex and costly. The proposed method in this paper avoids the complexity of route management by using a fixed sink and hierarchical routing. The summary and the highlights and limitations of each method in related work are shown in Table 1.

**Table 1:** The summary of all method in related work.

Designer’s name	Methodology	Advantages	Disadvantages
[5]	Combines the Firefly Algorithm and Fuzzy-Firefly Algorithm with the Random Forest technique for clustering in WSN	Increasing network lifetime	being trapped in local optima, and the RF needing labeled data for classifier training that may constrain the method’s applicability within WSNs
[6]	Clustering WSN using a Genetic Algorithm	Increasing the stability period, residual energy, network lifetime, and operational power.	High computational load.
[7]	Energy-aware clustering method using the Quantum Tunicate Swarm Algorithm.	The proposed method improves energy consumption while maximizing the WSN’s lifetime.	Using quantum bits to represent the solutions and applying quantum operators may introduce complexity and computational overhead to the algorithm
[8]	A new method for CH selection based on genetic algorithms and using optimal multi-hop routing	Enhancement in the network’s lifetime	Decentralized networks are more expensive and time-consuming to deploy because you need to install and configure multiple servers with load balancing and failover capabilities.
[9]	A new method for CH selection based on Moth Levy’s Artificial Electric Field Algorithm and data transfer-based Customized Grey Wolf Optimization.	Enhances the network lifetime	The electric field calculation algorithm involves mathematical complexities arising from integrations, coordinate transformations, or solving differential equations. Also, the Gray Wolf Optimization algorithm may experience low genetic diversity and get trapped in local optima during the early stages of execution.
[10]	Energy-aware clustering technique for data transmission in WSNs using the Harmony Search Algorithm with Competitive Swarm Optimization	It has a longer network lifetime.	The Harmony Search algorithm is heavily dependent on numerous parameters, such as the harmony memory size, pitch adjustment rate, and pitch changing rate, that significantly impact its performance.
[11]	Combining butterfly optimization and ant colony optimization to decrease energy consumption	It can minimize energy consumption, and extend the network’s lifetime.	This algorithm can be time-consuming and computationally burdensome.
[12]	A Grid-Based Clustering and Routing Algorithm for achieving energy-efficient routing in WSNs.	It can reduce the energy consumption at sensor nodes.	If the network size is too small, it may lead to the fragmentation of clusters into small pieces.
[13]	Clustering in Circular Networks: Using a systematic process to determine the optimal number of clusters based on the one-hop intra-cluster communication distance	Extends the scalability and the network’s lifetime.	CHs are exclusively selected based on the energy level criterion. Furthermore, due to the hierarchical transmission of all packets to the CHs closer to the sink, these nodes bear a higher communication load, resulting in quicker battery depletion and a hot-spot problem.
[14]	A distance-based energy-efficient algorithm.	Enhance energy consumption.	If the distance exceeds a specific threshold, an auxiliary node is employed to assist with data transmission. An auxiliary node, due to transferring the load of other nodes, bears a high energy burden.
[15]	A novel approach for CH selection in WSNs based on the remaining network energy exceeding a certain threshold (x(%	Increased network lifetime	Only the criterion of remaining energy has been considered for cluster selection.
[16]	Cluster-based client/server data aggregation.	Reduces energy consumption, decreases data aggregation delay, and improves the accuracy of data sampling.	In the proposed method, only the Hamming distance criterion is considered for selecting CHs.
[17]	A method for optimal clustering using the swarm intelligence of fish	Enhance energy efficiency.	The convergence speed may decrease in dynamic environments.
[18]	The genetic-based clustering method combined with the incorporation of the "onion" technique	Facilitates the creation of clusters and reduces communication burdens among CH nodes.	Higher computational expenses
[19]	Cluster-based routing method using a mobile sink	Enhance the network’s lifetime, energy consumption, end-to-end delay, and packet delivery ratio.	Determining the optimal path for the sink’s movement in the network is complex and costly.

## 3. Suggested method

In this section, a brief explanation of the scatter search algorithm is provided, followed by the proposed method for clustering sensor nodes.

Scatter search (SS) was introduced by Glover in 1977 as a method for integer programming [20]. In this algorithm, solutions are generated in a non-random manner to consider the characteristics of different components in the solution space.

The scatter search approach to exploring the search space is done in such a way that in this algorithm, a list of the best solutions is always maintained (referred to as the Reference Set). This list is initially created randomly at the beginning of the algorithm and then improved using a local search algorithm. In each iteration of the algorithm, several subsets of the top solutions are created using the Reference Set. Then, the members of each subset are combined with each other, generating a set of new solutions (recombined candidates). This set is improved by a local search operator, and the results of this improvement are maintained in the Candidate Setlist.

At the end of the main loop of the scatter search algorithm, the reference setlist is updated based on the candidate setlist. In the following, the details of clustering WSNs using the scatter search algorithm are explained. Considering that the sink node has no energy constraints, the proposed method is performed in a centralized manner inside the sink. Since each node knows the position of the sink (and obtains its own position through GPS), it calculates its distance to the sink according to Eq 1. Then, each node generates a "node information" message, which includes the sensor node’s identifier, the distance from the sensor node to the sink, and the remaining energy of the sensor node, and sends it towards the sink. After receiving these messages from the sensor nodes, the sink performs clustering using the scatter search algorithm.


$$D_{i}=\sqrt{{\left(xs-xi\right)}^{2}+{\left(ys-yi\right)}^{2}+{\left(zs-zi\right)}^{2}}$$ (1)

Where Di represents the distance to the sink, (xs, ys, zs) is the position of the sink, and (xi, yi, zi) is the physical position of sensor node i. In the proposed method, the nodes are randomly distributed in the simulation environment.

**Fig 1:**
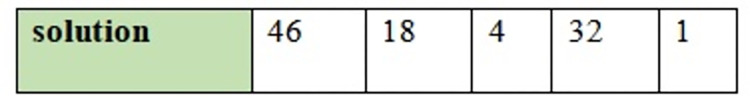
An example of a solution in the proposed method.

In the proposed method, clustering of sensor nodes is performed using the scatter search algorithm according to the following steps:

Step 1: Initialization of solutions: In this step, ten arrays of solutions (CH node identifiers) are randomly generated. For the fifty nodes present in the topology, five nodes will be selected as CHs randomly. Therefore, an initial array of five solutions is considered. Fig 1 illustrates an example of a solution where sensor nodes with IDs 46, 18, 4, 32, and 1 are randomly selected as CHs.

Step 2: Perform local search: In this step, the best solution (a solution with high fitness) is found using local search and placed inside the Reference Set, which always maintains a list of the best solutions. To calculate the best solution, for each node identifier listed in the solution, the fitness value based on two criteria—the remaining energy of each node and the distance to the sink—is calculated. To calculate the fitness for all sensor nodes in the solution, a fuzzy logic-based adaptive controller is utilized.

The employed fuzzy logic can provide precise results based on uncertain and ambiguous information [21].

In this paper, the Sugeno fuzzy model has been utilized to determine the fitnes,s of candidate CH nodes. In the design of the fuzzy model, two variables are considered fuzzy inputs: remaining energy and distance to the sink. The remaining energy (the first input) is the most crucial variable that directly impacts the network’s lifespan. If a node with low remaining energy decides to become a CH, it quickly depletes its energy and may not survive for long. The higher the remaining energy, the higher the priority for a node to become a CH.

The distance to the sink (the second input) determines the distance from the candidate CH node to the sink. The shorter this distance, the less energy is required to send data to the sink.

A Sugeno fuzzy rule with two inputs, x_1_ and x_2_, and one output, y, can be represented as "If a = x_1_ and b = x_2_, then f(x_1_,x_2_) = y," where a and b are the membership functions of the inputs, and y is the fitness or output value.

**Fig 2:**
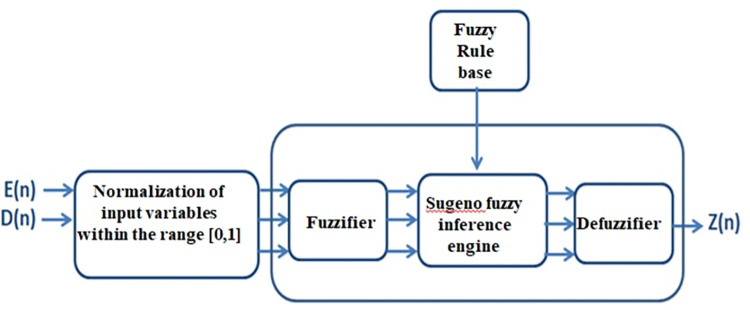
Block diagram of a fuzzy logic system.

The method for calculating the fitness of each node in the response list in the proposed pattern consists of four components (as depicted in Fig 2): normalization, fuzzification, the Sugeno fuzzy inference engine, and defuzzification. Additionally, as mentioned, two input variables, remaining energy (nE) and distance to the sink (nD), are considered for the nth node. The goal of the proposed method is to calculate the fitness of node n as a CH (nZ) based on these two input variables. The process involves normalizing the input variables, fuzzifying them to linguistic terms, applying the Sugeno fuzzy inference engine to determine the output fitness value, and finally defuzzifying the output to obtain a crisp value.

Considering the existence of different domains for each variable in each cluster, the input variables need to be normalized according to Eq 2 within the range of 0 to 1.


$$Normalized(X_{i})=\frac{X_{i}-Min(X)}{Max(X)-Min(X)}$$ (2)

In a way, xi represents the value of the input variable x for node i. Additionally, min(x) and max(x) denote the minimum and maximum values of the variable among all nodes within the cluster of node i.

**Fig 3:**
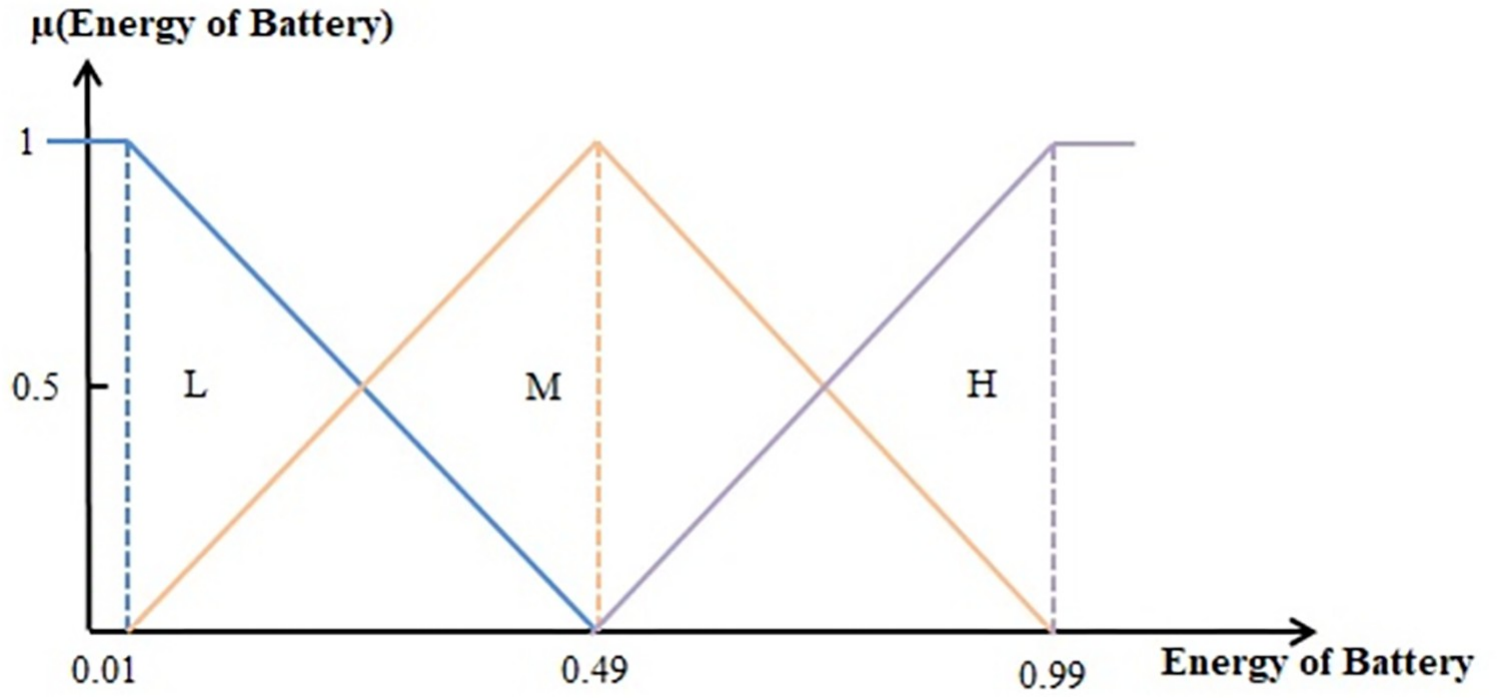
Membership functions for the input variable " Energy level of the sensor node’s battery.

**Fig 4:**
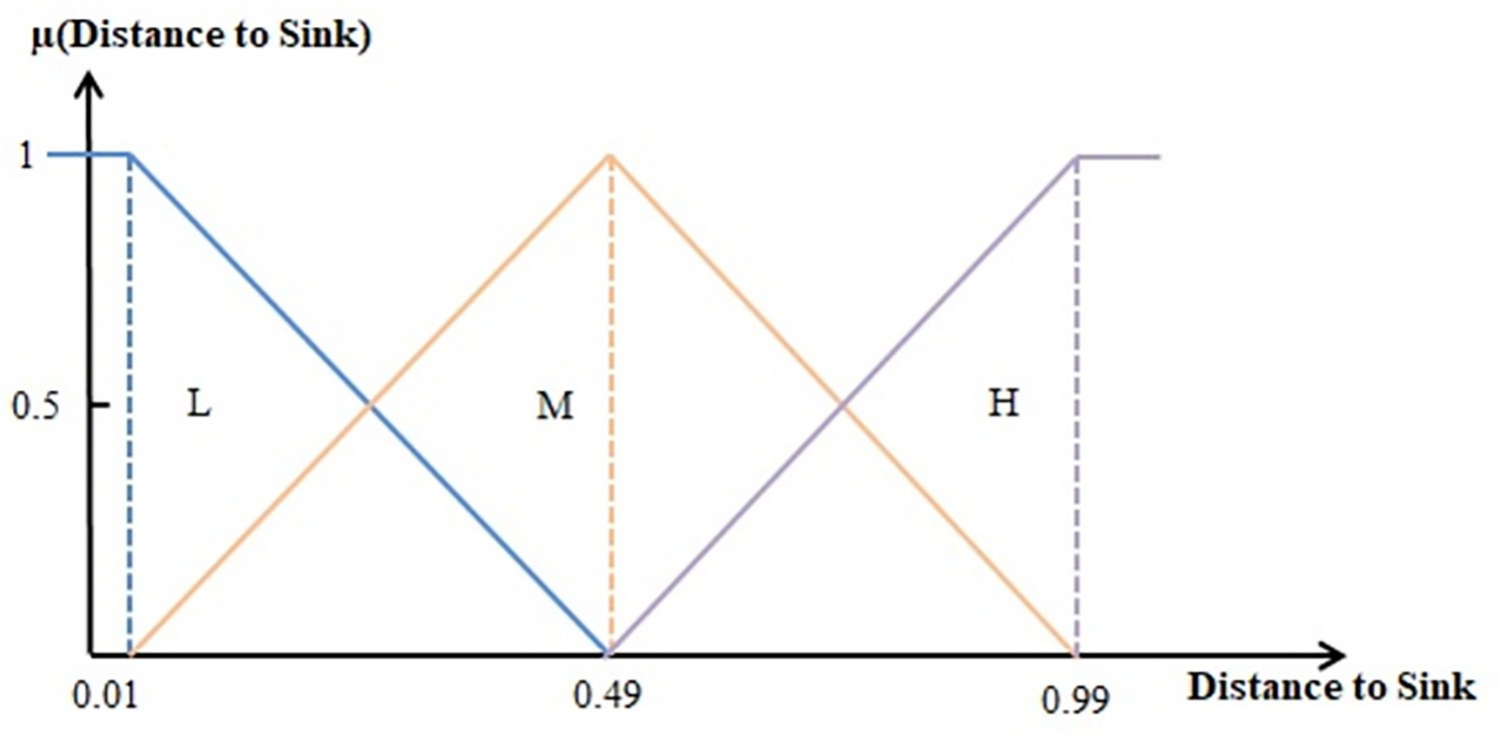
Membership functions for the input variable " Distance to sink".

**Fig 5:**
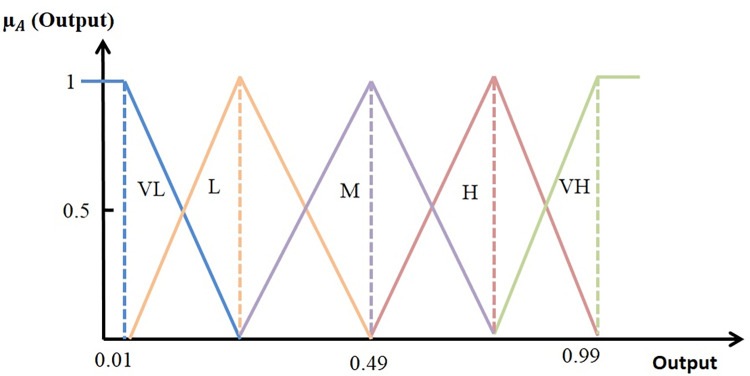
Membership functions for the output variable.

Fuzzifiers convert normalized inputs into linguistic fuzzy variables using membership functions. In this phase, fuzzy sets are defined for input variables, which include the distance of each sensor node to the sink and the energy level of the sensor node’s battery, as well as the fuzzy output, which represents the reliability of the sensor node. Three membership functions (H for high, M for medium, and L for low) are used for each input variable, as shown in Figs 3–5. Five membership functions (VH for very high, H for high, M for medium, L for low, and VL for very low) are used for the output, as depicted in Fig 5. After fuzzification, the fuzzy inference engine processes predefined Sugeno fuzzy rules.

In the inference stage, the reliability of each node is calculated using fuzzy rules and considering the values of the given parameters (node-to-sink distance and battery energy level). Each fuzzy rule consists of two parts: an antecedent part in the form of "if the node-to-sink distance is low and the battery energy is high" and a consequent part in the form of "then the node reliability will be very high."

For each of the input parameters, two fuzzy sets are defined, resulting in a total of nine fuzzy rules. These rules are defined in Table 2.

**Table 2:** Fuzzy rule base.

Rule	Input		Output
	Node-to-Sink Distance	Battery Energy	The fitness level of each sensor node.
R1	L	L	M
R2	L	M	L
R3	L	H	VL
R4	M	L	H
R5	M	M	M
R6	M	H	L
R7	H	L	VH
R8	H	M	H
R9	H	H	M

The fuzzy outputs for each activated rule are calculated. Finally, the defuzzifier aggregates the fuzzy outputs and converts them into a crisp value, denoted as Z(n), representing the fitness level of the node. Defuzzification: To translate the fuzzy outputs into numerical values, a defuzzifier is used. In this article, the centroid-of-gravity defuzzifier is employed, which is calculated using Eq 3.


$$Fitness\;Rate=\frac{\sum_{l=1}^{m}y^{-l}\prod_{i=1}^{n}\mu A_{i}^{l}(X_{i})}{\sum_{l=1}^{m}\prod_{i=1}^{n}\mu A_{i}^{l}(X_{i})}$$ (3)

where i is the index of the node, m is the number of fuzzy rules (in this case, 9), n is the number of membership functions for the input variables (in this case, 2), $\mu A_{i}^{l}(X_{i})$ is the fuzzy value of the membership functions, and *y*^−*l*^ is the output centers.

The fitness of each sensor node in the response list is determined by the fuzzy system. Eq 4 is used to calculate the fitness of the selected solution. According to this equation, the fitness of the solution array is the average fitness of all nodes whose IDs are listed.

In other words, the fitness of the solution is calculated as the average fitness of the nodes whose IDs are included in the list.

In fact, a node that is closer to the sink and has more energy will have a higher probability of becoming a CH (with high centrality), and its identifier will be included in the list of best responses.


$$Fit=\sum_{N}^{i=1}(Fitness\;Rate)/N$$ (4)

Fit refers to the fitness of a response, which is the fitness of the node whose identifier is included in the response list and is a candidate for becoming a CH. N represents the number of members in the response list. After calculating the fitness of each response, fifty percent of the responses with high fitness are selected. These selected responses are kept in the reference set list.

Step 3: Termination Condition Check: The termination condition is set to two hundred iterations of the algorithm. After two hundred iterations, the best responses (based on the highest fitness) are identified as CHs.

Step 4: Subgroup Formation: In each iteration, multiple subgroups of the top responses from the reference set list are created. Then, the combination operation is performed on each of these responses. A random number in the range of [1–5] is generated, and the combination operation is carried out from that point. The new responses are stored in the Recombined Candidates. The combination operation on a subgroup with two responses is illustrated in Table 3.

**Table 3:** An example of performing the fusion operation in the proposed method.

Refrence Set1	1	32	4	18	46
Refrence Set2	30	4	6	42	8
Recombined Candidate1	1	32	4	42	8
Recombined Candidate2	30	4	6	18	46

Step 5: Performing a local search: Once again, a local search is conducted on the list of new responses, namely the recombined candidates. The fitness of the new responses is calculated based on Eq 4, and fifty percent of the responses with high fitness are selected. The selected responses are then added to the candidateset list.

Steps 3 to 5 are repeated until the two hundredth iteration.

Step 6: Determining the clusters: In the two hundred iterations, the ReferenceSet list is updated based on the Candidateset list. Fifty percent of the responses with high fitness replace the responses with low fitness in the ReferenceSet list. Within the ReferenceSet list, the response with the highest fitness is selected, and its nodes are identified as the cluster centroids. Then, a notification packet indicating the cluster formation is generated, and the identifiers of the nodes in the best response in the ReferenceSet list are informed that they have become CHs.

Step 7: Connection Step: In this step, each sensor node that receives the CH notification message from the sink generates a message containing the CH node ID and the physical location of the CH (to announce the cluster formation) and broadcasts it within its range. Other nodes that are not CH nodes calculate their distances to the CH nodes and send connection messages to the nearest CH node. This process results in the formation of clusters as nodes establish connections with their respective nearest CH nodes.

**Fig 6:**
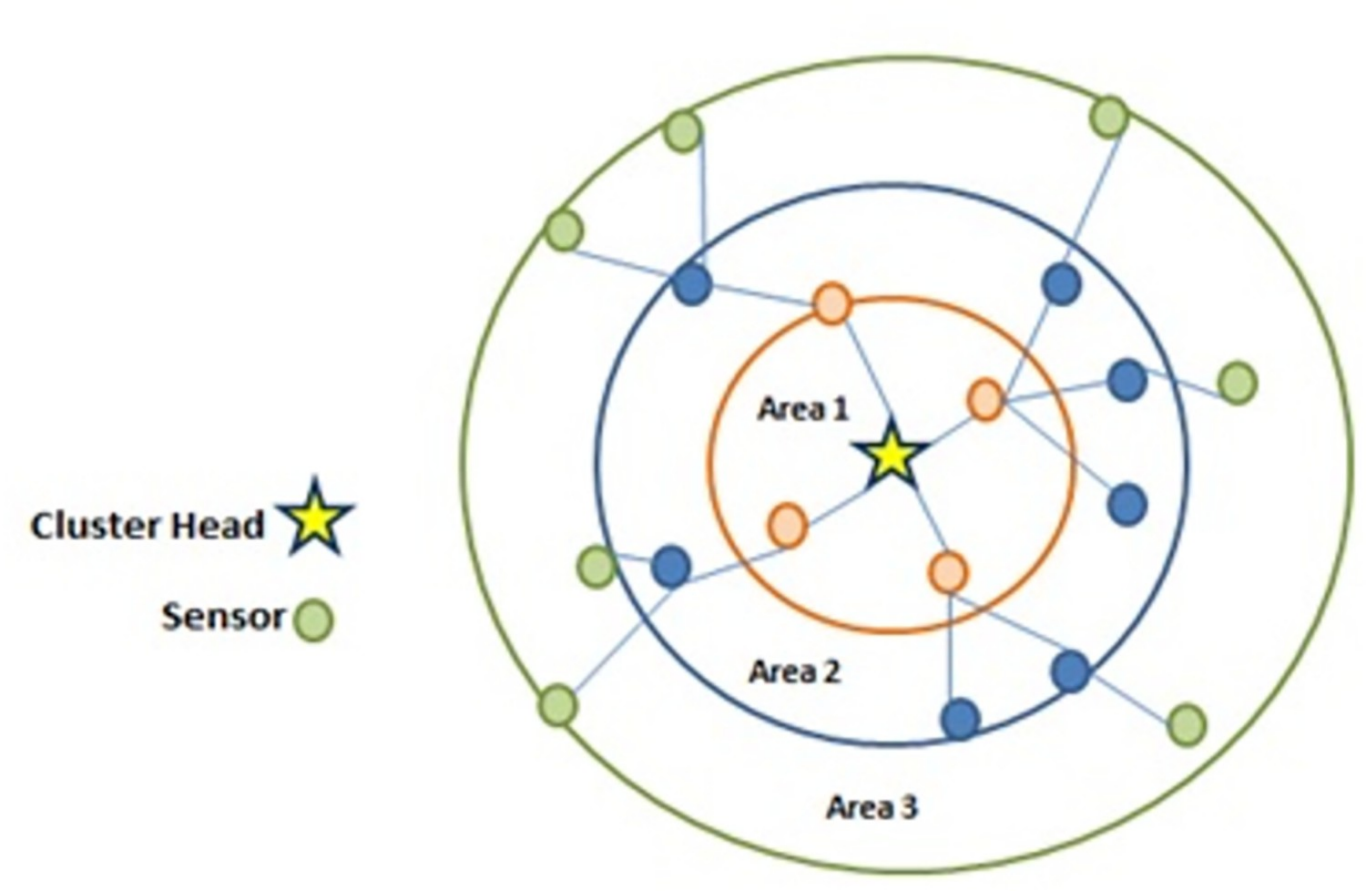
The process of creating the cluster-tree structure in the proposed method.

Step 8: To achieve a balance between energy consumption and data transmission delay, a multi-hop approach is employed. In this article, an adaptive multi-hop cluster-tree routing structure is proposed for the cluster member nodes. The cluster-tree routing structure reduces the data transmission distance from each sensor node to the CH and balances the network load within the cluster. As a result, the energy consumption of sensor nodes is reduced, and packet delays are kept at an ideal level. The process of creating the cluster-tree routing structure for the proposed scheme is illustrated in Fig 6.

Based on Fig 6, the non-CH nodes directly connect to the CH with the shortest distance in Zone 1. Then, the non-CH nodes in Zone 2, which have a shorter distance to Zone 1, connect to the non-CH nodes in Zone 1. The non-CH nodes in Zone 3 are connected to the non-CH nodes in Zone 2. This hierarchical connectivity pattern ensures that the nodes are connected in a cluster-tree structure, where the CH acts as the root of the tree and non-CH nodes are connected in a hierarchical manner based on their proximity to the CH and other non-CH nodes. In other words, the proposed data transmission method utilizes two techniques: intra-cluster data transmission and inter-cluster data transmission.

Intra-cluster data transmission: When a sensor node detects an event of interest, it transmits the data to its corresponding CH. If the CH is within the transmission range of the member nodes, direct communication takes place in a single-hop manner. However, if the CH is out of range, multi-hop communication occurs. This approach effectively conserves energy in the sensors and extends the overall network lifetime [22].

Inter-cluster data transmission: When cluster members transmit their data to the CH, the CHs forward the data to their nearest neighbor CH. This forwarding process continues until the data reaches the sink through the established data delivery path.

By employing these two methods, the proposed approach optimizes data transmission, enhances energy efficiency, and ensures the successful delivery of data throughout the network.

**Fig 7:**
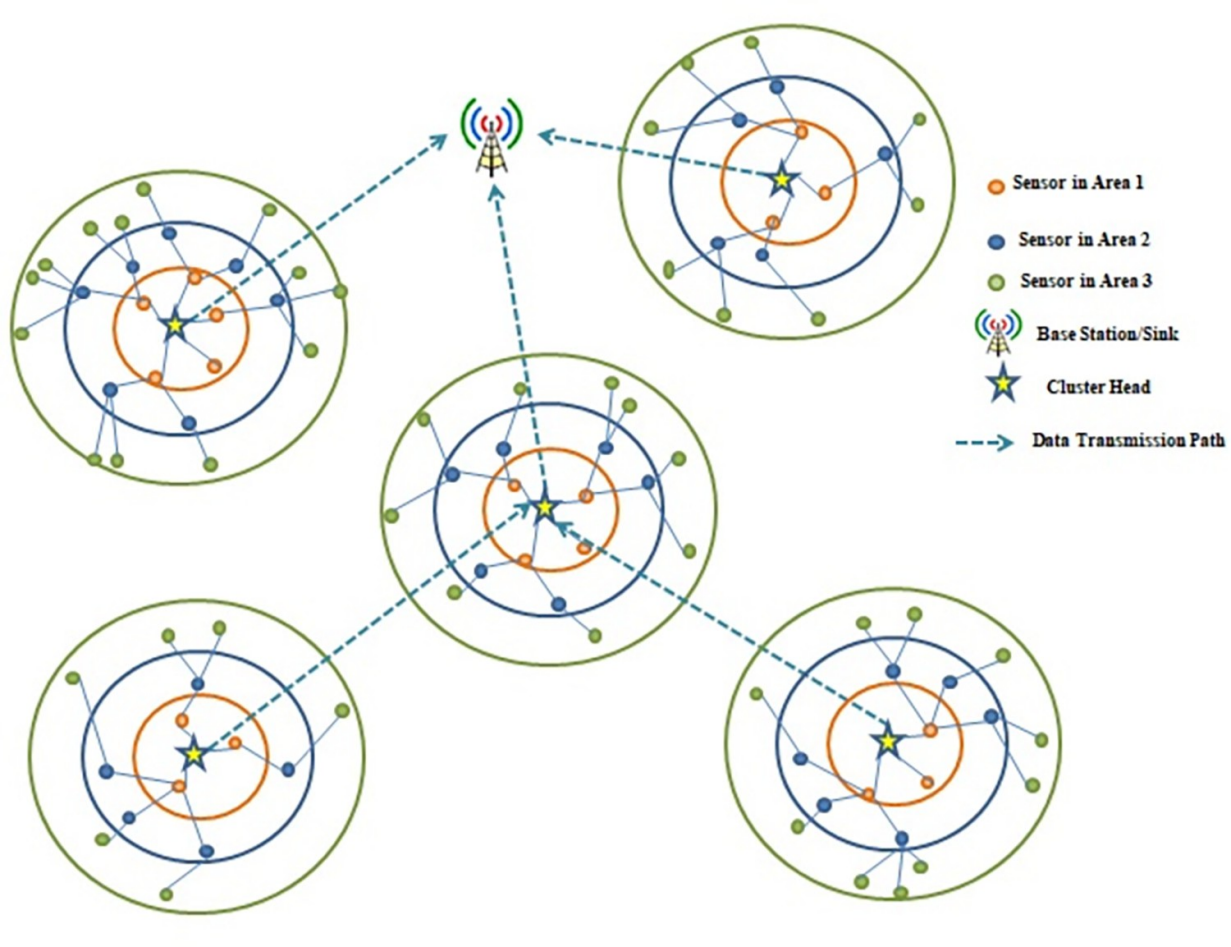
The network model for the proposed method.

The network model for the proposed method is illustrated in Fig 7.

**Fig 8:**
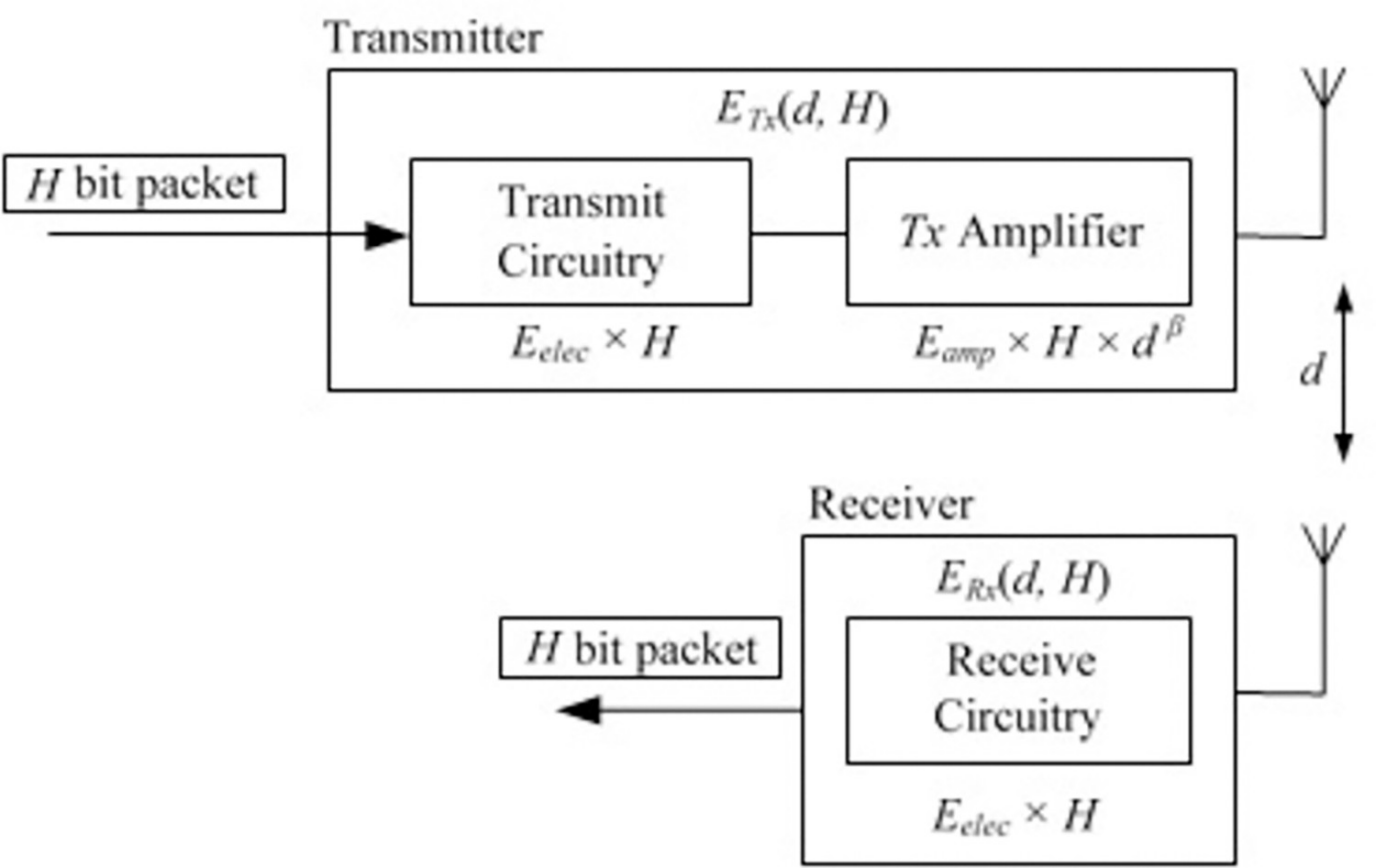
The energy consumption model [23].

The energy consumption model is depicted in Fig 8. Considering that energy consumption in radio transmission is typically higher than that in general sensor operations or memory access [23], we focus on the energy consumption for radio transmission and disregard the energy consumption for other operations.

In Fig 8 *E*_elec_ represents the energy dissipated per bit for the transmit or receive circuitry (measured in nano-Joules), *E*_*amp*_ indicates the energy consumption per bit for the power amplifier, and *β* denotes the path loss exponent. Therefore, when a transmitter sends an *H*-bit packet to a receiver, the total energy consumption can be calculated as according to Eq 5 [23].


$${E_{Tx}(d,H)=E_{elec}*H+E_{amp}*H*d}^{\beta }$$

$$E_{Rx}(d,H)=E_{elec}*H$$ (5)

Where *E*_*Tx*_ is the total energy consumption on transmitting, *E*_*Rx*_ is the total energy consumption on receiving an *H*-bit packet, respectively. Since all sensor nodes are assumed to be homogeneous, each sensor node starts with the same initial energy. However, the energy-rich sink is not subject to this energy consumption model; it presumably draws power from other abundant sources.

The overall operation of the scatter search algorithm can be seen in Algorithm 1.

Algorithm 1. Pseudocode for scatter search algorithm.

Function SS (problem) return a state that is a local optimum

Input: Problem_size_, DiverseSet_size_, ReferenceSet_size_

Output: S_best_

InitialSet ← ConstructInitialSolution (Problem_size_, DiverseSet_size_);

RefinedSet ←∅;

For S_i_ ∈ InitialSet do

RefineSet ←LocalSearch (S_i_);

end

ReferenceSet ← SelectInitialReferenceSet (ReferenceSet_size_);

While StopCondition () do

Subsets ← SelectSubset (ReferenceSet);

CandidateSet ← ∅

for Subset_i_ ∈ Subsets do

RecombinedCandidates ← RecombineMembers (Subset_i_);

for S_i_ ∈ RecombinedCandidates do

CandidateSet ← LocalSearch (S_i_);

end

end

ReferenceSet ← Select (RefernceSet, CandidateSet, ReferenceSet_size_);

end

**Fig 9:**
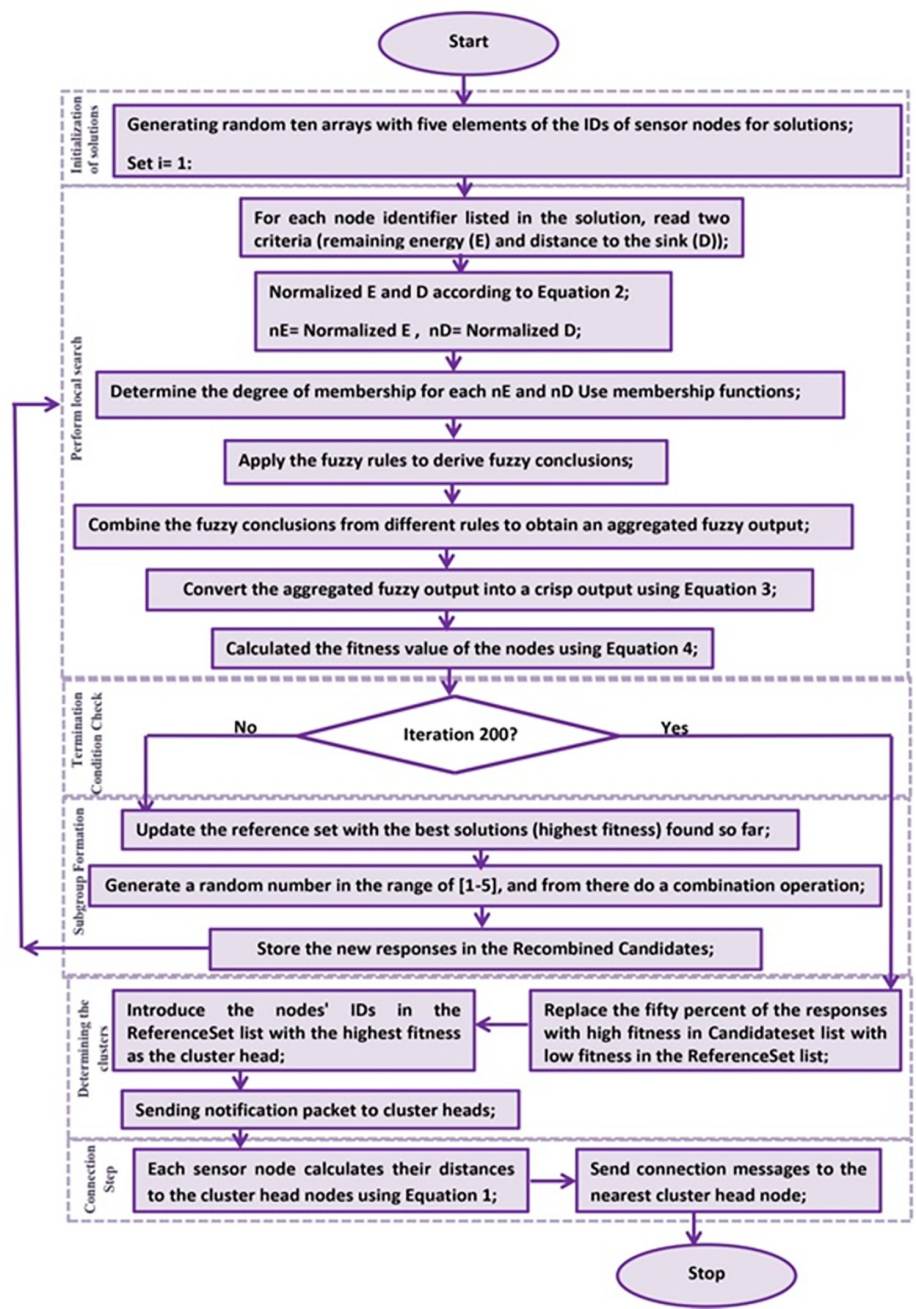
The general procedure of the proposed algorithm.

The general procedure of the proposed algorithm is shown in Fig 9.

## 4. Simulation of the proposed method

### 4.1. Simulation environment

**Fig 10:**
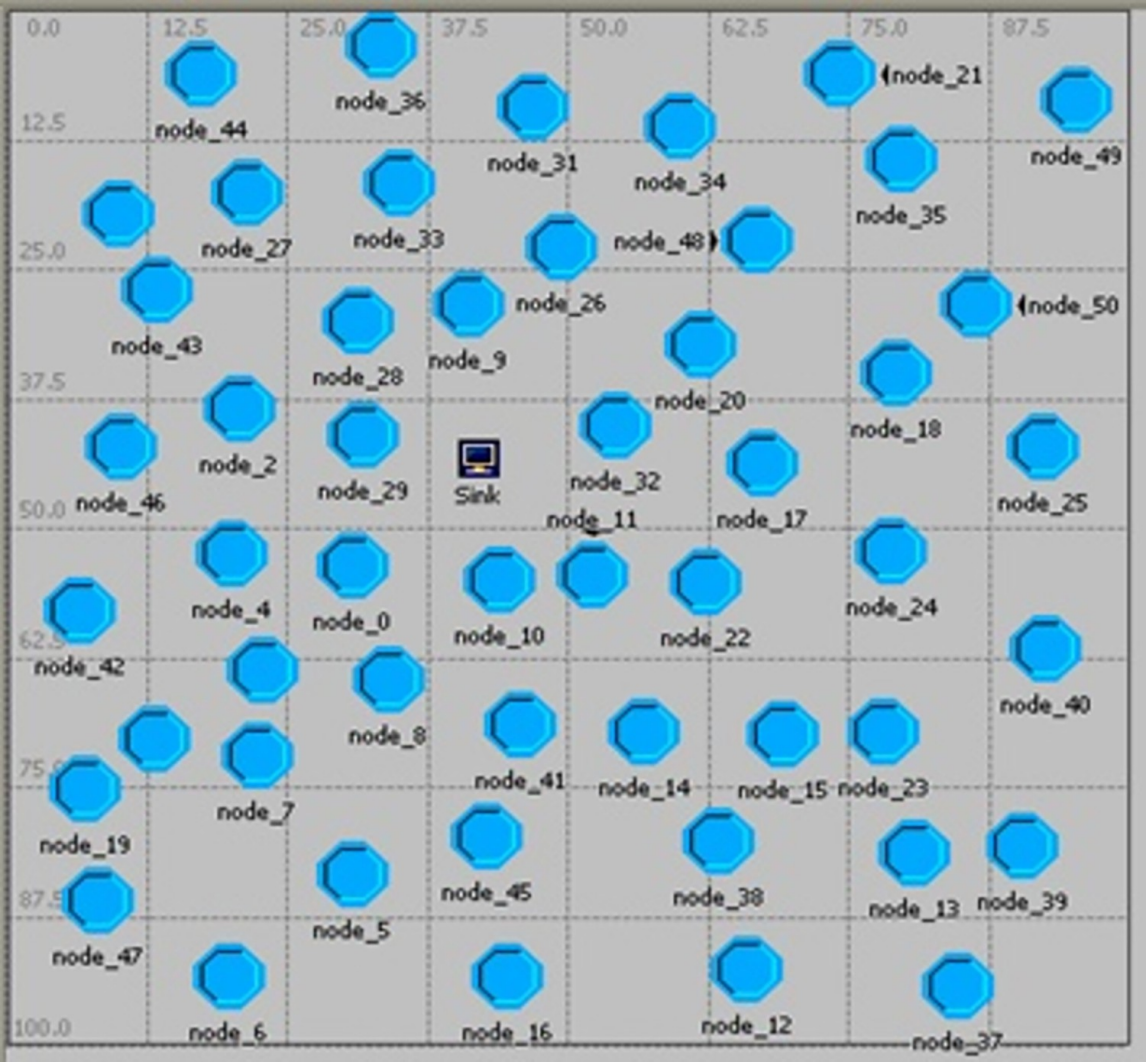
Network topology with 50 sensor nodes.

**Fig 11:**
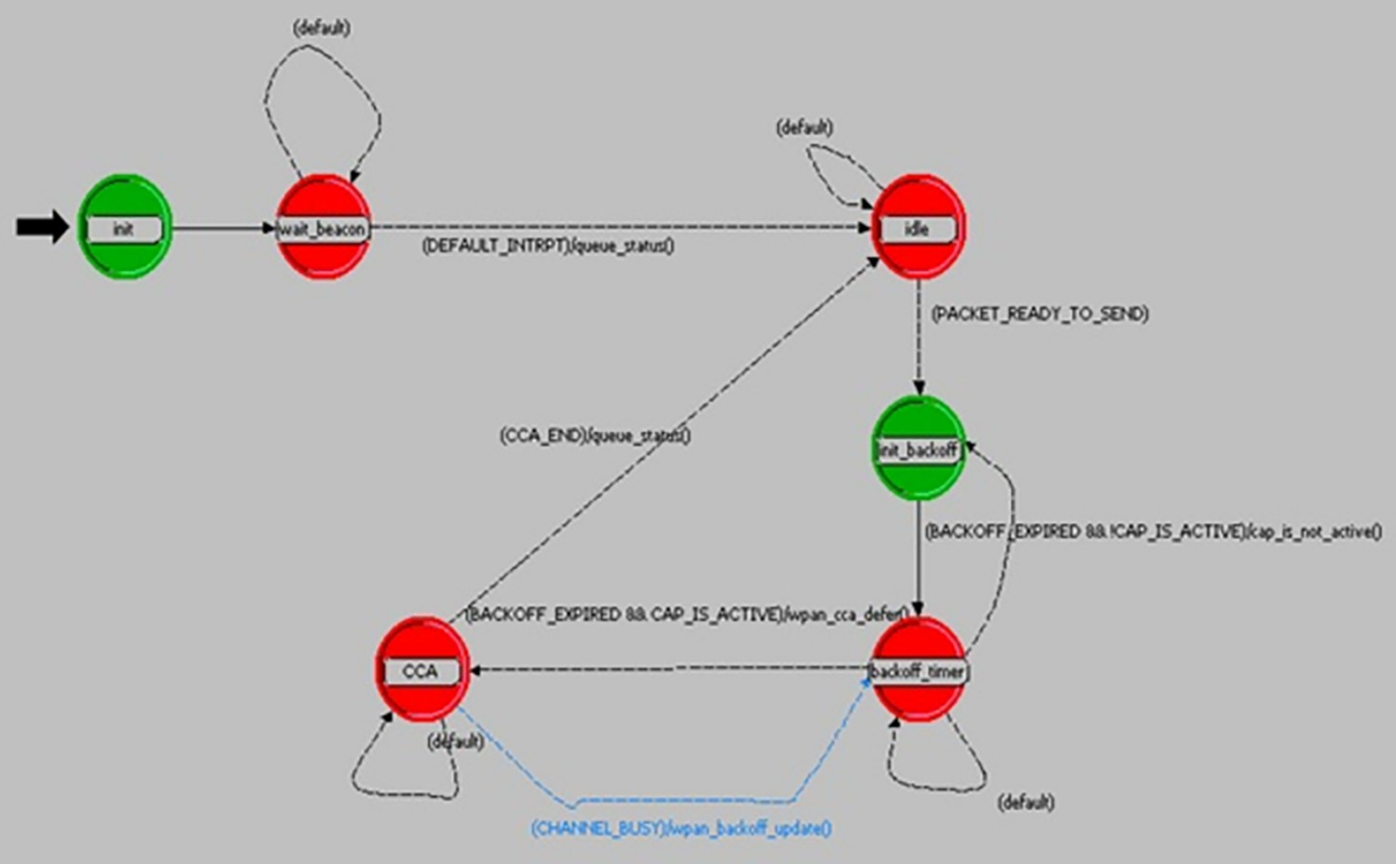
Proposed process model for sensor nodes.

In this paper, the Opnet Modeler software was used for simulating the proposed method and comparing it with the AFSRP protocol. Since Opnet Simulator encompasses a comprehensive collection of tools and models for accurately representing the behavior of WSNs, provides extensive features and capabilities for simulating these networks, and also includes realistic modeling of communication protocols, network topologies, and node behaviors, enabling detailed analysis of system performance, it can be considered the ideal choice for simulating WSNs. Table 4 presents the simulation parameters employed by this simulator. As mentioned, in one scenario, nodes are randomly deployed in a scattered environment based on the AFSRP protocol (a clustering protocol based on the fish swarm algorithm in WSNs). In the second (proposed) scenario, nodes are randomly deployed in a broadcast environment and are clustered using the proposed scatter search and fuzzy logic based clustering algorithm (SSFBCA) with the goal of improving energy consumption in routing. We will now discuss the simulation results of the proposed protocol based on these scenarios. It is worth noting that in both scenarios, a network topology with 50 MICAz sensor nodes was considered (as depicted in Fig 10). MICAz is easy to implement and can be used to build WSNs for various applications [24]. The process model, as shown in Fig 11, was utilized for the nodes in the topology.

**Table 4:** Simulation parameters.

Amount	Parameter
50	Number of nodes
250 Sec	Simulation time
100*100*100 m3	Simulation environment
250 m	Radio transmission board
1024bit	packet size
30 packets per second	Packet transmission rate
400 Joule	The initial amount of energy for all the sensor nodes
IEEE802.15.4	Mac layer protocol

### 4.2. Performance criteria in the proposed method

The following criteria are utilized to evaluate the efficiency of the proposed method: The following criteria are utilized to evaluate the efficiency of the proposed method:

#### 4.2.1. End-to-end delay

The average time it takes for a packet to be delivered from the source to the destination is referred to as the end-to-end delay. In the proposed method, end-to-end delay (*D*_*E*2*E*_) can be calculated according to Eq 6.


$$D_{E2E}=\frac{\sum_{i=1}^{n}\left(t_{arrive}^{i}-t_{send}^{i}\right)}{n}$$ (6)

**Fig 12:**
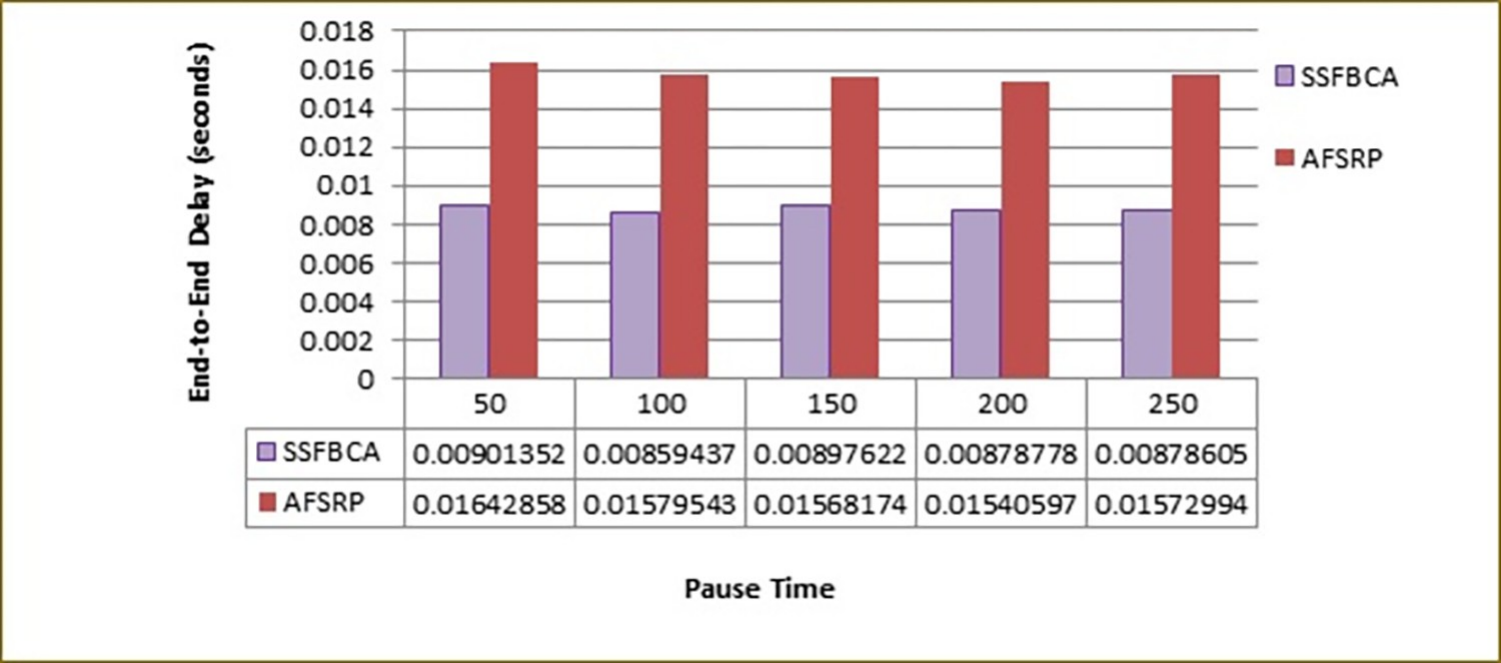
End-to-end delay.

Where n is the total number of packets sent from the source to the destination, $t_{arrive}^{i}$ is the time when the ith packet arrives at the destination, and $t_{send}^{i}$ is the time when the ith packet was sent from the source. Fig 12 compares the end-to-end delay for the proposed algorithm scenario (SSFBCA) and the AFSRP protocol scenario. The vertical axis represents the end-to-end delay, and the horizontal axis represents the simulation time. As observed, by employing the proposed protocol, the end-to-end delay rate for the network topology with 50 nodes improved by 13.44% compared to the AFSRP scenario. In the AFSRP protocol, due to unstable links (improper selection of CHs), data transfer may be incomplete, resulting in increased an end-to-end delay. However, in the proposed protocol, the utilization of the scatter search algorithm and the fuzzy control system for route selection leads to higher stability in the selection of CHs based on two parameters. Furthermore, the scatter search algorithm utilizes an experimental solution as an input, which promotes diversity. It also employs an optimization method to transform an experimental solution into an advanced experimental solution. These features enable the selection of optimal clusters compared to the AFSRP protocol, resulting in end-to-end delay reduction in the network. Furthermore, the scatter search algorithm utilizes an experimental solution as an input, which promotes diversity. It also employs an optimization method to transform an experimental solution into an advanced experimental solution. These features enable the selection of optimal clusters compared to the AFSRP protocol, resulting in an end-to-end delay reduction in the network.

#### 4.2.2. Media access delay

Media Access Delay refers to the duration between receiving a packet by the MAC layer and the complete loading of the packet onto the media. In the proposed method, Media Access Delay (D_MA_) can be calculated according to Eq 7.


$$D_{MA}=\frac{\sum_{i=1}^{n}\left(t_{load}^{i}-t_{receive}^{i}\right)}{n}$$ (7)

**Fig 13:**
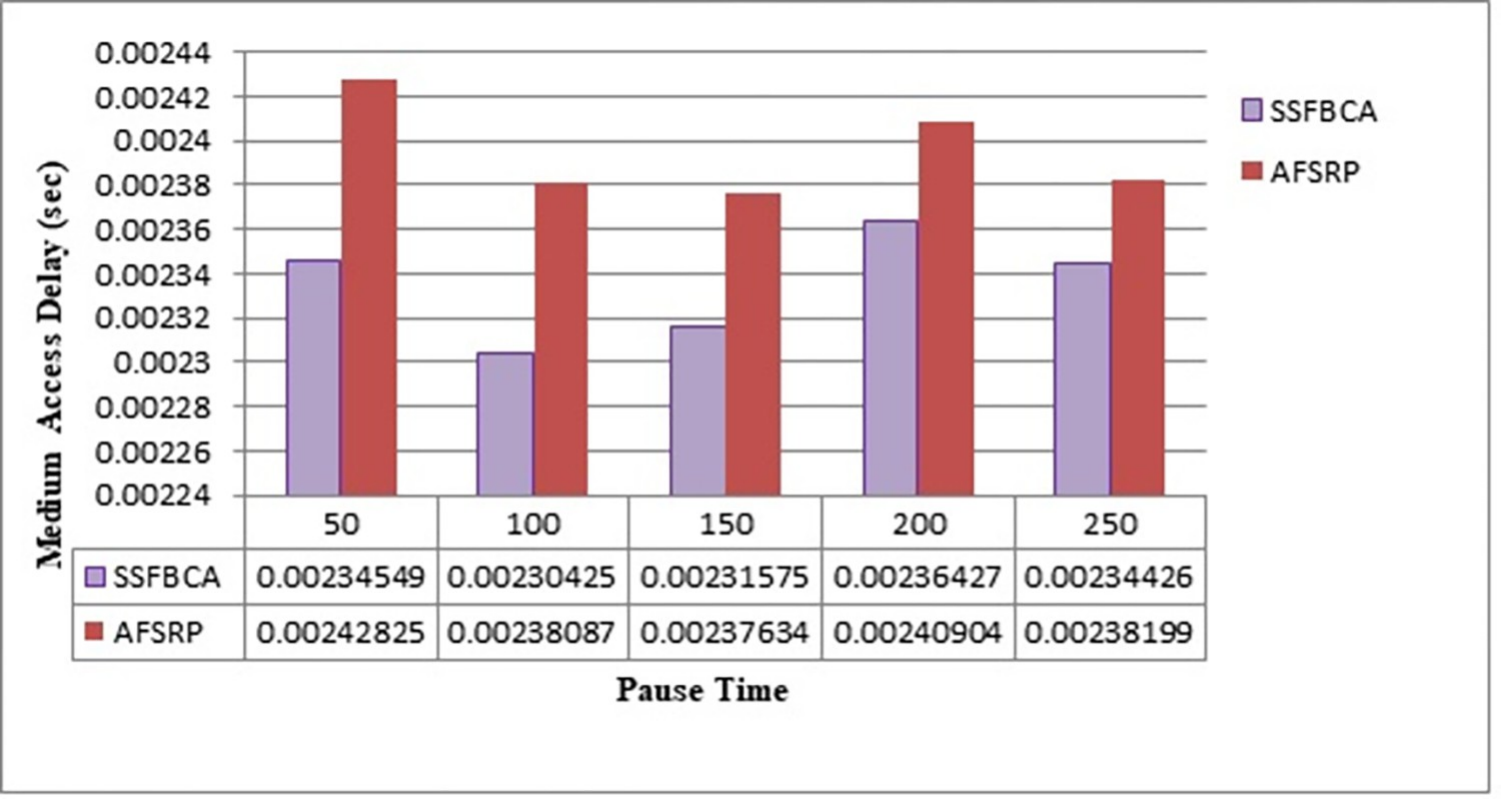
Media access delay.

Where n is the total number of packets received by the MAC layer, $t_{load}^{i}$ is the time when the ith packet is completely loaded onto the media and $t_{receive}^{i}$ is the time when the ith packet is received by the MAC layer. Fig 13 compares the media access delay for the proposed algorithm scenarios and the AFSRP protocol scenario. The vertical axis represents media access delay, and the horizontal axis represents simulation time. As observed, by employing the proposed protocol, the media access delay rate has improved by 75.2% for a network topology with 50 nodes compared to the AFSRP scenario. This issue arises from the fact that in the AFSRP protocol, the energy of cluster nodes may decrease, leading to the invalidation of the selected route. Consequently, data re-routing incurs additional delays. However, in the proposed protocol, the presence of a cluster-tree routing structure within each cluster reduces the data transmission distance from each sensor node to the CH, thus balancing the network load. Therefore, it is capable of effectively managing a high number of simultaneous access requests to the media. Furthermore, by reducing the number of requests and preventing congestion, it enhances efficiency and prevents congestion.

#### 4.2.3. Throughput

Throughput refers to the total number of received packets by the receivers divided by the time interval between receiving the first packet and the last packet. In the proposed method, throughput (Th) can be calculated according to Eq 8.


$$Th=\frac{N}{t_{last}-t_{first}}$$ (8)

**Fig 14:**
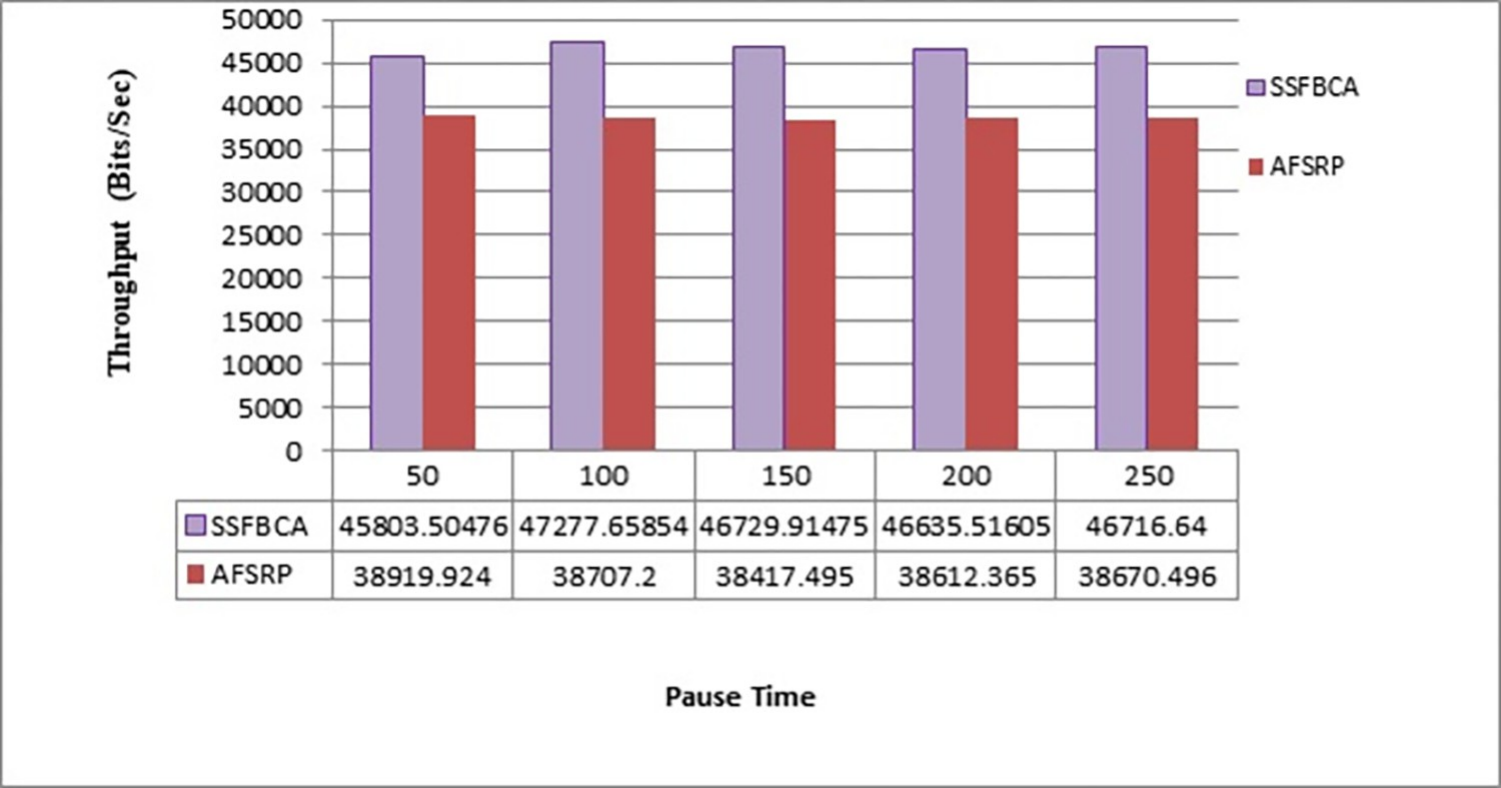
Throughput rate.

Where N is the total number of packets received by the receivers, t_last_ is the time when the last packet is received, and t_first_ is the time when the first packet is received. The throughput rates in the presented scenarios can be observed in Fig 14. The horizontal axis represents simulation time, and the vertical axis represents the throughput rate. As observed, by employing the proposed protocol, the throughput rate for a network topology with 50 nodes has improved by 20.55% compared to the AFSRP protocol scenario. This issue can be attributed to the intelligence of the proposed protocol for route selection. The protocol utilizes a scatter search algorithm and fuzzy logic to determine suitable CHs and sends data through these selected CHs. Additionally, the proposed method incorporates a cluster-tree routing structure within each cluster and sends data through the optimal path, which contributes to the improvement of the throughput rate. In the AFSRP protocol, the throughput rate is lower compared to the proposed method due to the occurrence of congestion and potential node failures. These factors can lead to reduced efficiency and lower throughput in the network.

#### 4.2.4. Packet delivery ratio (PDR)

PDR refers to the total number of data packets that are successfully transmitted from the sender sensor to the sink and received at the sink without any errors. In the proposed method, PDR can be calculated according to Eq 9.


$$PDR=\frac{N_{received}}{N_{sent}}*100\%$$ (9)

Where N_received_ is the total number of data packets successfully received at the sink and N_sent_ is the total number of data packets transmitted by the source node.

**Fig 15:**
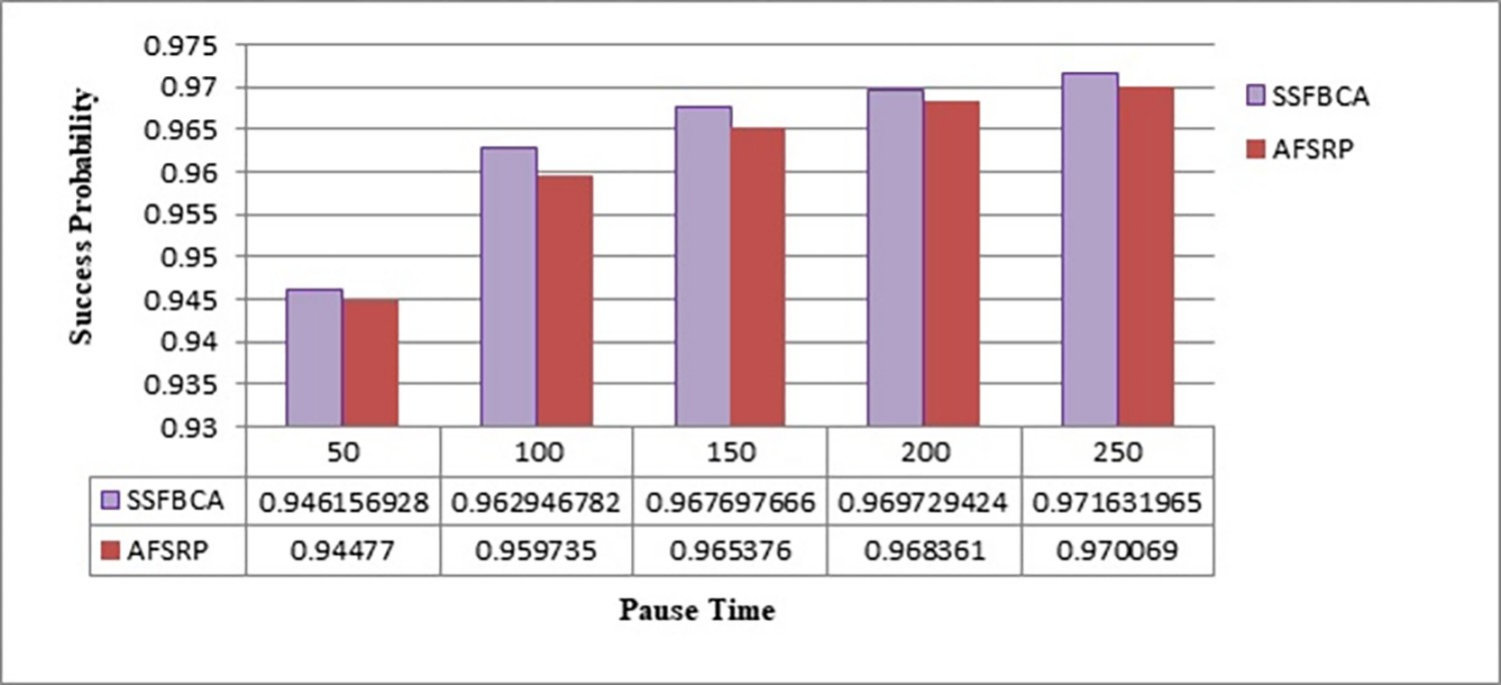
Packet delivery success rate.

Fig 15 illustrates the packet delivery ratio in the presented scenarios. The higher the packet delivery ratio, the better the system’s ability to successfully transmit data packets from the sender sensors to the sink without any errors. The horizontal axis represents simulation time, and the vertical axis determines the data packet delivery rate. As observed, by employing the proposed protocol, the data packet delivery rate has improved by 0.21% for a network topology with 50 nodes compared to the AFSRP protocol scenario. The inability to access a route towards the destination is one of the reasons for data packet loss in a network. Therefore, the proposed method aims to reduce data loss and increase the data delivery percentage by utilizing a scatter search algorithm, fuzzy logic, and selecting paths that remain stable until the end of the data transfer phase. In the AFSRP protocol, some network nodes may shut down due to battery depletion caused by high computations or data transmission. As a result, the data transfer to the sink node may not be completed, leading to a decrease in the success rate of data delivery.

#### 4.2.5. Signal-to-noise ratio (SNR)

SNR represents the ratio of the power of a signal to the power of the noise imposed on it. In the proposed method, SNR can be calculated according to Eq 10.


$$SNR=\frac{P_{signal}}{P_{noise}}$$ (10)

**Fig 16:**
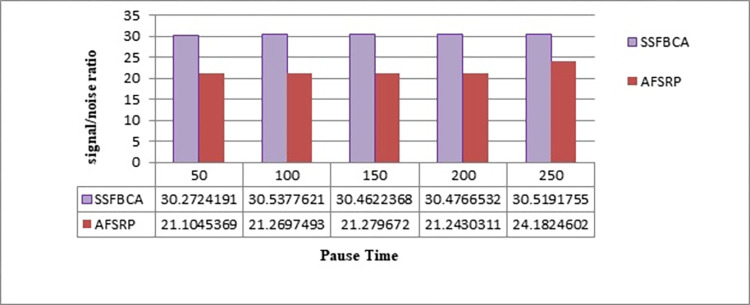
Signal-to-noise ratio.

Where P_signal_ is the power of the signal, and P_noise_ is the power of the noise. A higher SNR indicates a stronger signal relative to the noise, resulting in better signal quality and more reliable communication. Conversely, a lower SNR implies a weaker signal in comparison to the noise, which can degrade the quality of the signal and potentially cause communication errors or disruptions. In Fig 16, the graph represents the signal-to-noise ratio. The horizontal axis represents simulation time, and the vertical axis represents the signal-to-noise ratio. By employing the proposed protocol, the signal-to-noise ratio has improved by 43.40% for a network scenario with 50 nodes compared to the AFSRP protocol scenario. As observed, the AFSRP protocol has a lower signal-to-noise ratio compared to the proposed method. This is because in the AFSRP protocol, the number of error-prone bits during transmission may increase, leading to a decrease in the signal-to-noise ratio. Furthermore, in the AFSRP protocol, the transmitted signal may be lost or heavily corrupted due to congestion and interference. The probability of high noise occurrences is also increased. In the proposed method, due to the selection of suitable CHs by the scatter search algorithm and fuzzy logic, the likelihood of interference is reduced, leading to an increase in the signal-to-noise ratio.

#### 4.2.6. Average energy consumption of network nodes’ batteries

**Fig 17:**
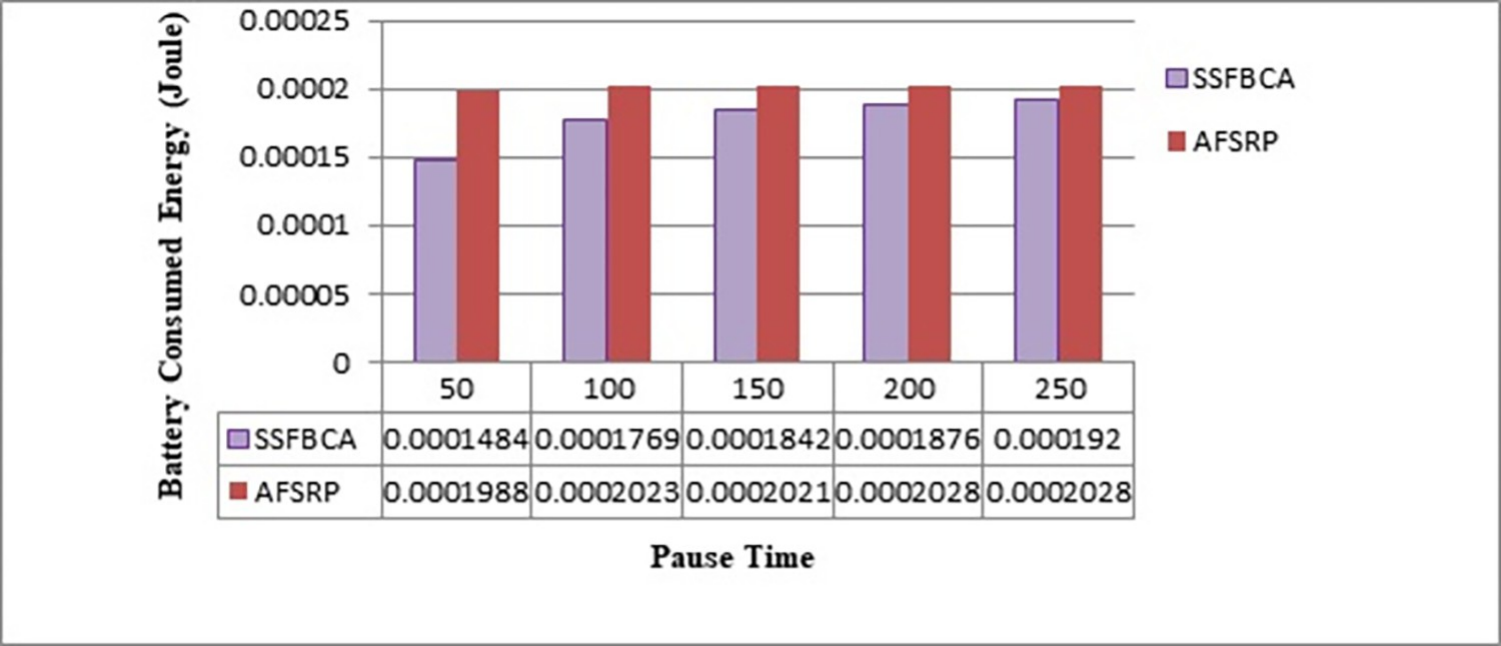
Energy consumption.

The average energy consumption of network nodes’ batteries refers to the total energy consumed by the nodes within the network for communication purposes, including data transmission. In the proposed method, the average energy consumption of network nodes’ nodes’ batteries is calculated according to Eq 5. Fig 17 illustrates the comparison of the average energy consumption of batteries for the proposed algorithm scenarios and the AFSRP protocol scenario. The vertical axis represents the average energy consumption, and the horizontal axis represents the simulation time. As observed, by employing the proposed protocol, the average energy consumption of batteries has improved by 13.52% for a network topology with 50 nodes compared to the AFSRP protocol scenario. In the AFSRP protocol, the node that is closer to the sink and has remaining energy below a threshold becomes the CH. If a node with such characteristics is not available, the algorithm does not perform re-clustering. As a result, the selected CHs in subsequent rounds may have lower energy and deplete their energy quickly, leading to network topology disruption. Additionally, in this protocol, CHs directly send the collected data to the sink node. Nodes that are far away from the sink will consume more energy to transmit data. In the proposed protocol, cluster formation and sink tracking are performed using the scatter search algorithm and fuzzy logic. A node is selected as a CH for data transmission and communication if it has higher energy, a shorter distance to the sink, and a shorter distance to the cluster center. Moreover, considering that member nodes join a CH or higher-level nodes based on their distance, there is no significant energy consumption required for data transmission from member nodes to the CH.

## 5. Conclusion

In this paper, energy consumption was studied as one of the most challenging issues in WSN. In order to improve energy consumption, an optimized algorithm based on scatter search and fuzzy logic with inputs such as battery energy levels and distance to the sink was proposed. The simulation results of the proposed protocol in OPNET were compared with the AFSRP routing protocol. Simulation results were extracted in the form of graphs to examine the performance of the proposed method. These graphs include energy consumption, signal-to-noise ratio, end-to-end delay, multimedia access delay, data packet delivery rate, and throughput. In general, it was observed that the proposed protocol exhibits better behavior compared to AFSRP. The proposed routing protocol, due to the selection of suitable nodes as CHs, chooses more stable routes for data transmission, thus improving the overall network performance. Furthermore, due to the utilization of a tree structure within each cluster, it leads to a reduction in energy consumption and a decrease in data transmission delay. These factors enhance the reliability of packet delivery and contribute to the scalability of the proposed protocol compared to the AFSRP protocol. In future work, the proposed method will be extended to operate in large-scale networks, and the network topology will be tested and evaluated with a higher number of nodes. Additionally, in future research, fault tolerance capabilities will be incorporated to handle errors occurring during operation.

## Supporting information

S1 File (ZIP)

## References

[1] Uthra R. A., & Raja S. V. (2012). QoS routing in wireless sensor networks—A survey. ACM Computing Surveys (CSUR), 45(1), 9.

[2] Al-Sulaifanie A. I., Al-Sulaifanie B. K., & Biswas S. (2022). Recent trends in clustering algorithms for wireless sensor networks: A comprehensive review. Computer Communications.

[3] Rana, S. N., & Kamboj, P. (2016, March). Resource utilization based congestion control for wireless sensor network: A review. In Computing for Sustainable Global Development (INDIACom), 2016 3rd International Conference on (pp. 715–720). IEEE.

[4] Sohrabi K., Gao J., Ailawadhi V., & Pottie G. J. (2000). Protocols for self-organization of a wireless sensor network. IEEE personal communications, 7(5), 16–27.

[5] Esmaeili H., Hakami V., Bidgoli B. M., & Shokouhifar M. (2022). Application-specific clustering in wireless sensor networks using combined fuzzy firefly algorithm and random forest. Expert Systems with Applications, 210, 118365.

[6] Sahoo B. M., Pandey H. M., & Amgoth T. (2022). A genetic algorithm inspired optimized cluster head selection method in wireless sensor networks. Swarm and Evolutionary Computation, 75, 101151.

[7] Srinivas P., & Swapna P. (2022). Quantum tunicate swarm algorithm based energy aware clustering scheme for wireless sensor networks. Microprocessors and Microsystems, 94, 104653.

[8] Kaedi M., Bohlooli A., & Pakrooh R. (2022). Simultaneous optimization of cluster head selection and inter-cluster routing in wireless sensor networks using a 2-level genetic algorithm. Applied Soft Computing, 128, 109444.

[9] Malisetti N., & Pamula V. K. (2022). Energy efficient cluster based routing for wireless sensor networks using moth levy adopted artificial electric field algorithm and customized grey wolf optimization algorithm. Microprocessors and Microsystems, 93, 104593.

[10] Kumar A., Webber J. L., Haq M. A., Gola K. K., Singh P., Karupusamy S., et al. (2022). Optimal cluster head selection for energy efficient wireless sensor network using hybrid competitive swarm optimization and harmony search algorithm. Sustainable Energy Technologies and Assessments, 52, 102243.

[11] Amutha J., Sharma S., & Sharma S. K. (2022). An energy efficient cluster based hybrid optimization algorithm with static sink and mobile sink node for Wireless Sensor Networks. Expert Systems with Applications, 203, 117334.

[12] Jannu, S., & Jana, P. K. (2014, April). Energy efficient grid based clustering and routing algorithms for wireless sensor networks. In Communication Systems and Network Technologies (CSNT), 2014 Fourth International Conference on (pp. 63–68). IEEE.

[13] Arghavani M., Esmaeili M., Esmaeili M., Mohseni F., & Arghavani A. (2017). Optimal energy aware clustering in circular wireless sensor networks. Ad Hoc Networks, 65, 91–98.

[14] Wang J., Li B., Xia F., Kim C. S., & Kim J. U. (2014). An energy efficient distance-aware routing algorithm with multiple mobile sinks for wireless sensor networks. Sensors, 14(8), 15163–15181. doi: 10.3390/s140815163 25196015 PMC4178983

[15] Abasıkeleş-Turgut İ., & Hafif O. G. (2016). NODIC: a novel distributed clustering routing protocol in WSNs by using a time-sharing approach for CH election. Wireless Networks, 22(3), 1023–1034.

[16] Ardakani S. P., Padget J., & De Vos M. (2016). CBA: A cluster-based client/server data aggregation routing protocol. Ad Hoc Networks, 50, 68–87.

[17] Gorgich S., & Tabatabaei S. (2021). Proposing an energy-aware routing protocol by using fish swarm optimization algorithm in WSN (wireless sensor networks). Wireless Personal Communications, 119(3), 1935–1955.

[18] Hatamian M., Barati H., Movaghar A., & Naghizadeh A. (2016). CGC: centralized genetic-based clustering protocol for wireless sensor networks using onion approach. Telecommunication systems, 62, 657–674.

[19] Ghorbani Dehkordi E., & Barati H. (2023). Cluster based routing method using mobile sinks in wireless sensor network. International Journal of Electronics, 110(2), 360–372.

[20] Glover, F. (1997, October). A template for scatter search and path relinking. In European conference on artificial evolution (pp. 1–51). Berlin, Heidelberg: Springer Berlin Heidelberg.

[21] Zadeh L. A. (1965). Fuzzy sets. Information and control, 8(3), 338–353.

[22] Jain S. K., Venkatadari M., Shrivastava N., Jain S., & Verma R. K. (2021). NHCDRA: a non-uniform hierarchical clustering with dynamic route adjustment for mobile sink based heterogeneous wireless sensor networks. Wireless Networks, 27, 2451–2467.

[23] Jiang J. A., Chen C. P., Chuang C. L., Lin T. S., Tseng C. L., Yang E. C., et al. (2009). CoCMA: Energy-efficient coverage control in cluster-based wireless sensor networks using a memetic algorithm. Sensors, 9(6), 4918–4940. doi: 10.3390/s90604918 22408561 PMC3291946

[24] Ali, N. A., Drieberg, M., & Sebastian, P. (2011, December). Deployment of MICAz mote for wireless sensor network applications. In 2011 IEEE International Conference on Computer Applications and Industrial Electronics (ICCAIE) (pp. 303–308). IEEE.

